# Knowledge on legislation of abortion and experience of abortion among female youth in Nepal: A cross sectional study

**DOI:** 10.1186/s12978-016-0166-4

**Published:** 2016-04-27

**Authors:** Ramesh Adhikari

**Affiliations:** Geography and Population Department, Mahendra Ratna Campus, Tribhuvan University, P.O.Box 1048, Kathmandu, Nepal

**Keywords:** Abortion, Legislation, Knowledge, Female, Youth, Nepal

## Abstract

**Background:**

Abortion has been legal in Nepal since 2002 and the country has made striking progress in rolling out induced abortion services. It led to well-known changes in reproductive behavior, however knowledge about legislation and abortion experience by female youth has been least investigated. This paper is an attempt to examine knowledge about legislation of abortion and abortion experiences among female youth in Nepal.

**Methods:**

This paper uses data from the Nepal Demographic and Health Survey (NDHS 2011). The analysis is confined to female youth aged 15-24 (*n* = 5050). Both bivariate and multivariate analyses have been performed to describe the knowledge about law and experience of abortion. The bivariate analysis (chi-square test) was applied to examine the association between dependent variables and female youth’s demographic, socioeconomic, and cultural characteristics. Besides bivariate analysis, the net effect of each independent variable on the dependent variable after controlling for the effect of other predictors has also been measured through multivariate analysis (logistic regression).

**Results:**

Only two-fifth (41 %) female youth was aware of abortion legislation in the country. Knowledge on at least one condition of abortion law is even lower (21 %). Less than two percent (1.5 %) female youth reported that they ever had an abortion. The multivariate analysis found that the knowledge and experience of abortion varied with different settings. Youth aged 20-24 [adjusted odds ratio (aOR) = 1.3; 95 % CI 1.7-5.0)], who have higher education (primary aOR = 1.89, ; 95 % CI 1.5-2.5 secondary aOR = 4.6; 95 % CI 3.7-5.9), who were from rich households (aOR = 1.5; 95 % CI 1.2-1.7), who had high autonomy (aOR = 1.29; 95 % CI 1.02-1.64) were more likely to be aware compared to their counterparts about legislation of abortion. In the other hand, female from Dalit (aOR = 0.55; 95 % CI 0.5-0.7 and Janajati aOR = 0.72; 95 % CI 0.6-0.8) caste, who were married (aOR = 0.80; 95 % CI 0.7-0.9), who were from Muslim (aOR = 0.54; 95 % CI 0.3-0.9) and who resided in Hill (aOR = 0.63 ; 95 % CI 0.5-0.8) and Terai/plain area (aOR = 0.74; 95 % CI 0.6-0.9) were less likely to be aware about the law. Similarly, female youth who have knowledge on abortion law (aOR = 2.8; ; 95 % CI 1.6-4.8), who have primary (aOR = 5.2; 95 % CI 1.6-16.9) and secondary education (aOR = 3.8; 95 % CI 1.2-12.8), married (aOR = 7.7; 95 % CI 3.8-12.9), who had higher number of children ever born [1-2 children aOR = 1.9; 95 % CI 1.1-3.6 and 3 or more children aOR = 3.4; 95 % CI 1.1-10.9), who were from rich households (aOR = 2.62 ;95 % CI 1.3-5.4), who have high autonomy (aOR = 3.0; 95 % CI 1.6-5.8), who had experienced sexual violence (aOR = 1.91; 95 % CI 1.1-8.7) were more likely to undergone abortion compared to their counterparts.

**Conclusion:**

Knowledge about legislation of abortion and conditions of abortion law is low among female youth. Awareness program should target these youth as they are more likely to be sexually active. There is a need of comprehensive education about abortion to these youth which can help eventually reduce unsafe abortion that take a large toll on women’s life.

## Background

Abortion has been legal in Nepal since 2002 and the country has made striking progress in rolling out induced abortion services, establishing comprehensive abortion care (CAC) at 100 % of public-sector sites at the regional, zonal, and district level and 46 % of primary health-care centers. As a result, more than 500,000 Nepali women have been served with safe abortion care since 2002 [1].

Worldwide, about 287,000 women die annually from pregnancy related causes [2]. Ninety percent of maternal deaths occur in less developed countries [3]. A large number of women (approx. 47000) died each year due to unsafe abortion [4]. It is also estimated that five million adolescents between the ages of 15 and 18 have unsafe abortion each year [5] and 70,000 abortion-related deaths occur among this age group every year [6]. In Nepal, the data suggest that more than a quarter (26 %) of all pregnancies [7] and 41 percent of the last pregnancy among currently pregnant women [8] are unintended.

About a fifth of the total population of Nepal comprise youth (between the ages of 15-24) [9]. While a majority of adolescents (64 %) have their first sexual intercourse between 15 to 17 years of age, only 4.5 percent of adolescent women use a modern form of contraception [10]. Less than two-fifth adolescent aged 15-19 thought that abortion is legal in the country. Less than three percent pregnancy had abortion before the age of 20 years [11]. Overall, unsafe abortion remains a leading cause of maternal morbidity and mortality in Nepal, accounting for an estimated 54 % of gynecological and obstetric hospital admissions and 42 % of maternal deaths in health sites [12].

Knowledge about abortion law among female youth is very important because it has implications for access to legal abortion services. Even when safe, legal abortion services are available, women who lack accurate information about the law may seek unsafe abortion because they do not know that they are eligible for the service or do not know the legal requirements for obtaining an abortion [13]. It is hypothesized that older female, who have higher education, who have higher level of autonomy in household decision, lead to high knowledge of abortion law and higher experience of abortion.

Research on awareness of abortion law and experience of abortion among female youth may help to inform policy makers and education planners in Nepal. Unfortunately, not much research has been conducted in this area among the female youth in the country. The aims of this study are to investigate awareness levels and factors influencing awareness of the abortion legislation among female youth in Nepal. Furthermore this study also explores the prevalence of abortion incidence among female youth in Nepal.

## Methods

Data for this paper were drawn from the Nepal Demographic and Health Survey, 2011. The primary purpose of the 2011 NDHS, a nationally representative sample survey,was to provide current and reliable data on fertility and family planning, child mortality, children’s nutritional status, utilization of maternal and child health services, domestic violence, and knowledge of HIV/AIDS. The 2011 NDHS was carried out under the aegis of the Population Division of the Ministry of Health and Population.

Interviews were completed for 12,674 women of reproductive age. The detail methods can be found in the NDHS report. [11] However, this analysis is confined to youth aged 15-24 women (*n* = 5050). Association between exploratory variables and knowledge on abortion law was assessed via bivariate analysis using chi-square tests. Then logistic regression was used to assess the net effect of each exploratory variable on knowledge on abortion law after controlling for several other independent variables.

## Results

Majority of the respondents were adolescent aged 15-19 (55 %). Almost two thirds had secondary or above education. Similarly, more than a half of youth were married. An overwhelming majority of youth lived in rural area (86 %). Almost three-fourth had no autonomy in household decision. Less than five percentages of female youth had experienced of physical, emotional and sexual violence by their husband/partner (Table 1).

**Table 1:** Background characteristic of female youth

	%	N
Age group		
15-19	54.5	2753
20-24	45.5	2297
Ethnicity		
Brahmin/Chhetri	31.9	1611
Janajati	40.6	2053
Dalit	15.1	762
Other	12.4	625
Education level		
No education	17.1	866
Primary	17.6	887
Secondary or above	65.3	3298
Marital status		
Never married	49.0	2476
Ever married	51.0	2575
Number of children ever born (CEB)		
None	65.7	3318
1-2	30.4	1534
3 or more	3.9	198
Religion		
Hindu	83.8	4231
Buddhist	9.1	458
Muslim	4.1	207
Kirat/Christian	3.1	155
Ecological zone		
Mountain	6.3	316
Hill	39.5	1996
Terai	54.2	2738
Place of residence		
Urban	13.7	692
Rural	86.3	4358
Wealth index		
Poor	35.5	1794
Middle	21.7	1095
Rich	42.8	2162
Autonomy in household decision		
No autonomy	72.6	3667
Moderate autonomy (involved in 1-2 issues)	16.0	809
High autonomy (involved in all 3 issues)	11.4	575
Physical violence by husband/partner		
No	96.0	4847
Yes	4.0	204
Emotional violence by husband/partner		
No	97.0	4898
Yes	3.0	152
Sexual violence by husband/partner		
No	97.7	4936
Yes	2.3	115
Total	100.0	5050

Only two fifths female youth (41 %) were aware about the law. However, only a fifth (21 %) was aware at least one condition of abortion law (Table 2). Awareness about abortion legislation varied with different settings. A significantly higher proportion of youth from Brahmin/Chhetri (55 %) than Janajati (43 %) and Dalit (28 %) were aware about the law. Similarly, significantly higher proportions of the youth who had secondary or above education (52 %) than those who had primary (26 %) or no education (13 %) were aware about the law. It is found that a higher percentage of never married youth (45 %) compared with ever married (37 %) were aware of abortion legislation. Similarly, a significant proportion of youth who lived in urban area, who were from rich family, who did not experience physical, emotional and sexual violence were aware about the law than other groups. In regards to experience of abortion, significantly higher youth aged 20-24, who had primary level education, who were ever married, who were from rich family, who had high autonomy, who had experienced sexual violence had abortion than other group (Table 3).

**Table 2:** Knowledge about abortion law and conditions

	%	N (5050)
Abortion is legal in Nepal		
Yes	41.0	2070
No	34.1	1720
Don’t know	25.0	1261
Conditions of Abortion law		
Pregnancy of 12 weeks or less gestation for any woman	13.0	659
Pregnancy of 18 weeks if it is a result of rape or incest	7.9	401
Pregnancy of any duration if life of mother is at risk	2.8	143
Pregnancy of any duration if mother’s physical and mental health at risk	3.8	192
Fetus is deformed	2.3	115
*At least one condition of abortion law*	*21.0*	*1059*

**Table 3:** Knowledge of legalization of abortion law and experience of abortion by background characteristic of female youth

	Knowledge on abortion law (%)		Have an abortion (%)		Total N
	No	Yes	No	Yes	
Age group	-			***	
15-19	60.2	39.8	99.5	0.5	2753
20-24	57.7	42.3	97.2	2.8	2297
Ethnicity		***		-	
Brahmin/Chhetri	45.4	54.6	98.4	1.6	1611
Janajati	57.4	42.6	98.3	1.7	2053
Dalit	72.3	27.7	98.4	1.6	762
Other	83.2	16.8	99.5	0.5	625
Education level of youth		***		**	
No education	86.8	13.2	99.6	0.4	866
Primary	74.0	26.0	97.7	2.3	887
Secondary or above	47.7	52.3	98.4	1.6	3298
Marital status		***		***	
Never married	54.6	45.4	100.0	0.1	2476
Ever married	63.3	36.7	97.1	2.9	2575
Number of children ever born (CEB)		***		***	
None	56.6	43.4	99.5	0.5	3318
1-2	61.6	38.4	96.3	3.7	1534
3 or more	79.0	21.0	97.4	2.6	198
Religion		***		-	
Hindu	57.5	42.5	98.5	1.5	4231
Buddhist	60.3	39.7	98.2	1.8	458
Muslim	90.7	9.3	99.4	0.6	207
Kirat/Christian	54.8	45.2	98.4	1.6	155
Ecological zone		*		-	
Mountain	54.3	45.7	99.0	1.0	316
Hill	57.9	42.1	98.4	1.6	1996
Terai	60.4	39.6	98.5	1.5	2738
Place of residence		***		-	
Urban	52.0	48.0	97.6	2.4	692
Rural	60.1	39.9	98.6	1.4	4358
Wealth index		***		***	
Poor	69.1	30.9	99.1	0.9	1794
Middle	64.4	35.6	99.2	0.8	1095
Rich	47.9	52.1	97.6	2.4	2162
Autonomy in household decision		-		***	
No autonomy	59.7	40.3	99.6	0.4	3667
Moderate autonomy (involved in 1-2 issues)	58.3	41.7	96.9	3.1	809
High autonomy (involved in all 3 issues)	55.9	44.1	93.8	6.2	575
Physical violence by husband/partner		***		-	
No	58.1	41.9	98.5	1.5	4847
Yes	81.2	18.8	97.8	2.2	204
Emotional violence by husband/partner		***		-	
No	58.5	41.5	98.5	1.5	4898
Yes	77.2	22.8	96.9	3.1	152
Sexual violence by husband/partner		***		*	
No	58.5	41.5	98.5	1.5	4936
Yes	79.4	20.6	96.0	4.0	115
Total	59.0	41.0	98.5	1.5	5050

Note *** Significant at Chi-square test *p* < 0.001, ** = *p* < 0.01, and * = *p* < 0.05

Logistic regression was conducted to examine the net effect of exploratory variables on awareness on abortion law. Crude and adjusted odds ratio are presented in the Tables 4 and 5. It is found that the variables age, ethnicity, education, marital status, religion, ecological zone, wealth index, autonomy in household decision were strong predictors for having awareness on abortion law. Those youth who had an abortion were almost 3 times more likely (aOR = 2.94; 95 % CI 1.72-5.0) to be aware of abortion law compared with those who never had an abortion. Similarly, respondents aged 20-24 were more likely to be aware about law (aOR = 1.28; 95 % CI 1.11-1.43) compared with respondents aged 15-19. Respondents from Janajati and Dalit were 28 percent and 45 percent respectively less likely to be aware about law compared with respondents from Brahmin/Chhetri. Similarly, youth who have secondary and above education were about 5 times more likely (aOR = 4.6, 95 % CI 3.65-5.92) than those who had no education. On the other hand, ever married female were less likely to be aware on abortion law (aOR = 0.80; 95 % CI 0.66-0.98) than unmarried female. Female from rich household were more likely to be aware about law (aOR = 1.5; 95 % CI 1.23-1.74) than female from poor household. Female who had high autonomy in household decision were more likely to be aware about law than those who did not have autonomy in household decision (Table 4).

**Table 4:** Adjusted odds ratio (aOR) and 95 % confidence interval (CI) for having awareness on abortion law of the country among female youth by selected predictors

Predictors	Crude			Adjusted		
	OR	95 % CI		OR	95 % CI	
		Lower	Upper		Lower	Upper
Had an abortion						
No	1.00			1.00		
Yes	3.6***	2.2	5.9	2.938***	1.724	5.004
Age group						
15-19	1.00			1.00		
20-24	1.11	0.99	1.24	1.283**	1.105	1.489
Ethnicity						
Brahmin/Chhetri	1.00			1.00		
Janajati	.616***	.540	.702	.719***	.619	.836
Dalit	.318***	.264	.384	.552***	.450	.678
Other	.168***	.134	.212	.332***	.250	.441
Education level						
No education	1.00			1.00		
Primary	2.3***	1.796	2.945	1.894***	1.460	2.457
Secondary or above	7.18***	5.835	8.848	4.651***	3.654	5.920
Marital status						
Never married	1.00			1.00		
Ever married	0.69***	0.62	0.78	.801*	.658	.975
Number of children ever born (CEB)						
None	1.00			1.00		
1-2	.812**	.718	.919	1.155	.946	1.410
3 or more	.348***	.246	.493	1.081	.706	1.654
Religion						
Hindu	1.00			1.00		
Buddhist	0.89	0.73	1.08	.875	.700	1.093
Muslim	0.14**	0.86	0.22	.540*	.312	.934
Kirat/Christian	1.12	0.81	1.54	.976	.694	1.373
Ecological zone						
Mountain	1.00			1.00		
Hill	.863	.680	1.096	.632**	.487	.819
Terai	.777*	.615	.982	.744*	.568	.973
Place of residence						
Urban	1.00			1.00		
Rural	0.72***	0.61	0.85	1.104	.916	1.331
Wealth index						
Poor	1.00			1.00		
Middle	1.235**	1.053	1.448	1.067	.892	1.276
Rich	2.426***	2.128	2.765	1.467***	1.234	1.743
Autonomy in household decision						
No autonomy	1.00			1.00		
Moderate autonomy (involved in 1-2 issues)	1.060	.908	1.237	1.015	.823	1.250
High autonomy (involved in all 3 issues)	1.169*	1.092	1.396	1.295*	1.023	1.640
Physical violence by husband/partner						
No	1.00			1.00		
Yes	.320***	.224	.457	.543	.266	1.108
Emotional violence by husband/partner						
No	1.00			1.00		
Yes	.416***	.284	.610	.923	.481	1.771
Sexual violence by husband/partner						
No	1.00			1.00		
Yes	.367***	.233	.579	.768	.390	1.512
Constant				.297***		
-2 Log likelihood				6023.9		
Cox & Snell R Square				.149		

Note *** *p* < 0.001, ** = *p* < 0.01, and * = *p* < 0.05

**Table 5:** Adjusted odds ratio (aOR) and 95 % confidence interval (CI) for having had an abortion among female youth by selected predictors

Predictors	Crude			Adjusted		
	OR	95 % CI		OR	95 % CI	
		lower	Upper		lower	Upper
Knowledge on abortion law						
No	1.00			1.00		
Yes	3.626**	2.204	5.967	2.771***	1.610	4.771
Age group						
15-19	1.00			1.00		
20-24	5.883***	3.242	10.675	1.142	.589	2.214
Ethnicity						
Brahmin/Chhetri	1.00			1.00		
Janajati	1.082	.649	1.804	1.374	.768	2.459
Dalit	.986	.495	1.965	1.428	.668	3.055
Other	.303*	.092	.993	.392	.082	1.861
Education level						
No education	1.00			1.00		
Primary	5.655**	1.818	17.587	5.186**	1.584	16.978
Secondary or above	3.973*	1.349	11.694	3.823*	1.146	12.752
Marital status						
Never married				1.00		
Ever married	8.356	5.378	13.227	7.709***	3.762	12.964
Number of children ever born (CEB)						
None	1.00			1.00		
1-2	8.243***	4.663	14.569	1.957*	1.051	3.644
3 or more	5.698**	2.064	15.729	3.462**	1.092	10.970
Religion						
Hindu	1.00			1.00		
Buddhist	1.175	.564	2.448	1.154	.503	2.647
Muslim	.370	.059	2.298	2.374	.226	24.890
Kirat/Christian	1.073	.303	3.805	.903	.237	3.439
Ecological zone						
Mountain	1.00			1.00		
Hill	1.569	.500	4.928	1.144	.343	3.811
Terai	1.456	.469	4.521	1.094	.324	3.689
Place of residence						
Urban	1.00			1.00		
Rural	.572*	.330	.993	.702	.375	1.313
Wealth index						
Poor	1.00			1.00		
Middle	.945	.412	2.169	.982	.406	2.374
Rich	2.871***	1.619	5.089	2.617**	1.254	5.460
Autonomy in household decision						
No autonomy	1.00			1.00		
Moderate autonomy (involved in 1-2 issues)	7.539***	3.999	14.216	1.624	.828	3.183
High autonomy (involved in all 3 issues)	15.281***	8.375	27.881	3.005**	1.568	5.759
Physical violence by husband/partner						
No	1.00			1.00		
Yes	1.508	.577	3.938	.481	.108	2.133
Emotional violence by husband/partner						
No	1.00			1.00		
Yes	2.160	.839	5.557	.827	.184	3.708
Sexual violence by husband/partner						
No	1.00			1.00		
Yes	2.833*	1.084	7.402	1.906*	1.017	8.722
Constant				0.01***		
-2 Log likelihood				595.17		
Cox & Snell R Square				.038		

Note *** *p* < 0.001, ** = *p* < 0.01, and * = *p* < 0.05

Female youth who had knowledge on abortion law were almost 3 times (aOR = 2.8; 95 % CI 1.61-4.77) more likely to have an abortion than those who were not aware about the law. Similarly, those who have primary (aOR = 5.2; 95 % CI 1.58-16.9) and secondary or above (aOR = 3.8, 95 % CI 1.15-12.75) education, who had higher number of children (aOR = 1.9; 95 % CI 1.05-3.64 for those who had 1-2 children and aOR = 3.5, 95 % CI 1.09-10.97 for those who had 3 or more), who had higher autonomy in household decision (aOR = 3.01; 95 % CI 1.56-5.76) were more likely to have an abortion than their comparison group. It is found that women who had experienced of sexual violence from their husbands/partners were about twice (aOR = 1.9; 95 % CI 1.02-8.72) more likely to have an abortion than those who did not face sexual violence from their intimate partners (Table 5).

## Discussion

This study explored the awareness level and factors influencing awareness about abortion legislation among female youth in Nepal and their experience on abortion. In order to ensure that legalized abortion in Nepal improves reproductive health, people must know that abortion is a legal option in case of unintended pregnancy. Research shows that most youth had experienced unprotected sex and unintended pregnancy [8, 14]. In such cases, awareness about abortion law could play a critical role in reducing unintended birth and unsafe abortion.

Safe abortion is an effective means of preventing unintended birth, but unfortunately the large numbers of youth are unaware of it. The awareness of law among female youth was 41 %, which is low compare to study conducted in South African women (69 %) [15], slightly higher than Mexican youth (45 %) [16] and Latvia (57 %) [17].

Similarly, our study found that older youth were more aware about the law. It could be due to that older youth feel comfortable to talk about reproductive health issues than younger female. On the other hand, female youth from Janajati and Dalit were less likely to be aware about the law compared with Brahmin/Chhetri.

As expected, youth with higher education were more likely to be aware about the abortion law than those who did not have education. This could be due to these students could talk the issues such as about family planning methods, abortion, or sex-related issues with their friends and colleague without reluctant [18]. However, married youth were less likely to be aware about the abortion law than those unmarried.

Ecological differences in awareness level can be found in this study. Youth from hill and terai region were less likely to be aware about the abortion law than those who live in mountain region. Those who were rich and who had higher level of autonomy were more likely to be aware about the law than their comparison group. However, this finding also requires further investigation. 

There are some limitations in the interpretation of the results of this study. First, as pointed out previously, we restricted our subjects to only female youth, so our results regarding the awareness about the abortion law and the prevalence of abortion incidence should not be generalized to all women in Nepal. Second, because the cross-sectional design of the study and all of the items analyzed in the logistic regression analysis came from information at the time of survey, the analysis can only provide evidence of statistical association between those items and knowledge on abortion law and cannot show cause-effect relationships.

## Conclusion

Awareness about abortion legislation among female youth was low which indicates that there is a need among female youth for information on abortion. Health education initiatives should target such youth as they are more likely to be sexually active. It can help to reduce unintended birth, many of which result in unsafe abortion and take a large toll on women’s health.
